# Agreement, Reliability, and Feasibility of Melbourne Rapid Fields Tablet Perimetry Compared with Humphrey Field Analyzer in Glaucoma

**DOI:** 10.3390/diagnostics16213528

**Published:** 2026-10-27

**Authors:** Murshidah Hassan Basri, Teck Chee Cheng, Rona Asnida Nasaruddin, Jemaima Che Hamzah

**Affiliations:** 1 Department of Ophthalmology, Faculty of Medicine, Universiti Kebangsaan Malaysia, Jalan Yaakob Latiff, Bandar Tun Razak, Cheras, Kuala Lumpur 56000, Malaysia; musyeehb123@gmail.com (M.H.B.); eddiecheng88@gmail.com (T.C.C.);; 2 Department of Ophthalmology, Hospital Canselor Tuanku Muhriz, Jalan Yaakob Latiff, Bandar Tun Razak, Cheras, Kuala Lumpur 56000, Malaysia

**Keywords:** test–retest reliability, Melbourne Rapid Fields (MRF), Humphrey Field Analyzer (HFA), glaucoma, visual field indices

## Abstract

**Objectives**: We aimed to evaluate the agreement, test–retest reliability, test duration, and patient acceptability of Melbourne Rapid Fields (MRF) tablet perimetry compared with Humphrey Field Analyzer (HFA) 24-2 SITA-Standard perimetry in glaucoma patients. **Methods**: This cross-sectional study evaluated 64 eyes of 64 patients with primary or secondary glaucoma. Participants underwent both HFA and MRF perimetry in a non-randomized sequence. Agreement was analyzed using intraclass correlation coefficients (ICC) and Bland–Altman analysis. Test–retest reliability of MRF was evaluated in 47 participants after 4 weeks. Acceptability was assessed via questionnaires. **Results**: MRF demonstrated comparable reliability indices to HFA (*p* > 0.05), although these findings do not establish equivalent clinical reliability due to differences in platform-specific definitions. MRF demonstrated excellent test–retest reliability for MD (ICC 0.993), PD (ICC 0.992), and VC (ICC 0.992; all *p* < 0.001). Compared with HFA, MRF produced significantly less negative MD (−6.27 ± 5.55 vs. −8.32 ± 5.47 dB, *p* < 0.001) and higher PSD/PD (7.48 ± 3.26 vs. 6.33 ± 4.11 dB, *p* < 0.001), while VFI/VC did not differ significantly (*p* = 0.808). Overall agreement was good-to-excellent MD (ICC 0.949), PSD/PD (ICC 0.880), and VFI/VC (ICC 0.930). However, exploratory severity-stratified Bland–Altman analysis suggested greater variability in moderate and severe glaucoma. MRF was significantly faster than HFA (4.22 ± 0.81 vs. 5.46 ± 1.33 min, *p* < 0.001). All patients reported it was easy and comfortable. **Conclusions**: MRF provides excellent test–retest reliability, shorter testing duration, and high patient acceptability. While overall agreement is high, systematic differences and greater advanced-stage variability prevent direct interchangeability with HFA. Instead, MRF serves as a practical, complementary platform for accessible, frequent testing.

## 1. Introduction

Glaucoma is a chronic optic neuropathy characterized by progressive retinal ganglion cell loss, structural optic nerve changes, and visual field defects [1,2], representing the second leading cause of blindness worldwide and an important cause of irreversible visual impairment [3]. Because functional loss may progress despite apparently stable clinical findings, visual field is fundamental for glaucoma diagnosis and longitudinal monitoring. Standard automated perimetry (SAP), most commonly performed using the Humphrey Field Analyzer (HFA; Carl Zeiss Meditec, Jena, Germany), is widely regarded as the reference standard for assessing glaucomatous visual field loss [2]. HFA measures differential retinal sensitivity at predefined locations by presenting stimuli of varying luminance against a standardized background [4].

Despite its established clinical role, SAP is affected by test–retest variability, which makes distinguishing true progression challenging—especially in slow progressors [5]. More frequent SAP testing can shorten the time required to identify statistically significant visual field deterioration, particularly in eyes with faster rates of functional loss [6]. However, frequent testing is difficult to achieve due to high cost, requiring dedicated space and trained personnel, and is non-portable.

Several strategies have been developed to improve the efficiency, including faster thresholding algorithms incorporated into HFA and alternative perimeters such as frequency-doubling technology (FDT), head-mounted perimeter, Medmont and Compass [7,8,9]. Although these technologies have demonstrated acceptable reliability and reproducibility [8,10], HFA remains the primary reference standard against which newer perimetric technologies are evaluated. Comparative studies have demonstrated differences in mean deviation (MD), pattern standard deviation (PSD) and point-wise sensitivity between alternative perimeters and HFA, highlighting that agreement with conventional SAP needs to be established before adopting new technologies [7,8,9,11].

The increasing availability of portable digital devices has created an opportunity to address some of these limitations. Tablet-based perimetry uses high-resolution displays to deliver visual stimuli, potentially allowing testing outside standard perimeter environments. Several applications have been developed, including Visual Field Easy, MyScotoma, Performance-Centered Portable Test (PERCEPT) and Melbourne Rapid Fields (MRF) [12], and have been investigated for clinic-based screening, monitoring and home-based assessment [12,13]. While reviews highlight their potential to enhance testing frequency and accessibility, clinical evidence remains heterogeneous, and conventional SAP remains the benchmark against which they must be validated [13,14].

Among these, Melbourne Rapid Fields (MRF) is an iPad-based application (Glance Optical Pty Ltd., Melbourne, Australia) designed for portable threshold perimetry using a Bayesian estimation strategy [15]. Initial validation demonstrated that MRF provides quantitative threshold measurements across the central visual field [15]. Subsequent studies show good correlation and repeatability between MRF and HFA, though systematic differences in global indices and measurement variability have been reported [16,17,18,19,20].

The first comparative study of MRF and HFA in glaucoma patients demonstrated strong correlations for global visual field indices and suggested that MRF could provide a portable alternative where conventional perimetry was not readily available [17]. Schulz et al. subsequently evaluated MRF in glaucoma patients, pre-perimetric glaucoma and healthy controls and demonstrated good agreement with HFA together with acceptable sensitivity for detecting glaucomatous defects [18]. A six-month longitudinal study further demonstrated good repeatability of MRF measurements and showed that MRF required less testing time than HFA SITA-Standard [19]. More recently, Kumar et al. reported similar findings in an Indian glaucoma population, with MRF demonstrating shorter test duration but significant differences in MD and PSD/PD compared with HFA [20]. These studies provide encouraging evidence for MRF, but they also demonstrate that agreement does not necessarily imply interchangeability between the two testing platforms.

Importantly, most of the available evidence evaluating MRF has originated from Australian or other non-Malaysian populations. Population-specific differences in disease severity, visual field characteristics, testing behaviour and familiarity with digital technology may influence the performance and acceptability of a tablet-based test. Furthermore, previous studies have varied considerably in their study populations, testing protocols and assessment of reliability. A recent systematic review of home-based perimetry also highlighted the limited evidence for diagnostic accuracy and the methodological heterogeneity of existing studies [14]. Thus, further validation of portable perimetry in different clinical populations and healthcare settings remains necessary.

In addition to agreement with conventional SAP, test–retest reliability and patient acceptability are important considerations for a portable visual field test intended for repeated use. This is particularly relevant if tablet perimetry is ultimately to be used for community-based or home monitoring, where patients may perform testing with less supervision and under less controlled conditions. Early studies of home-based perimetry have demonstrated promising feasibility and adherence, but have also identified challenges related to patient engagement, fixation, testing environment and device handling [19,21].

In this study, we evaluated the Melbourne Rapid Fields (MRF) tablet-based perimetry application using an 11-inch Apple iPad Pro and compared its visual field results with those obtained using HFA 24-2 SITA-Standard [22] in patients with primary and secondary glaucoma in a Malaysian tertiary ophthalmology centre. We specifically assessed agreement of global perimetric indices, test–retest reliability, test duration and patient acceptability. By evaluating these parameters within a Malaysian clinical population, we aimed to determine whether MRF could provide a reliable and practical adjunct to conventional SAP and to identify the clinical settings in which portable tablet perimetry may have potential value.

## 2. Materials and Methods

### 2.1. Subjects

This cross-sectional observational study was conducted from 1 February 2020 to 31 March 2021 at the Ophthalmology Clinic, Hospital Canselor Tuanku Muhriz, Malaysia. Consecutive patients with primary or secondary glaucoma who fulfilled the eligibility criteria were recruited. The study was approved by the Medical Research and Ethics Committee of Universiti Kebangsaan Malaysia (JEP-2019-829) and was conducted in accordance with the Declaration of Helsinki and the Malaysian Guidelines for Good Clinical Practice. Written informed consent was obtained from all participants before enrolment. Permission to use the Melbourne Rapid Fields (MRF) application for research purposes was obtained from Glance Optical, including a non-commercial agreement. The MRF application was installed on an 11-inch iPad Pro and registered using the investigator’s institutional email account.

Participants were eligible if they were aged ≥18 years, had a best-corrected visual acuity of 20/40 or better, had previous experience with standard automated perimetry using the Humphrey Field Analyzer (HFA; Carl Zeiss Meditec, Inc., Dublin, CA, USA), and were able to understand and follow test instructions in English. For HFA testing, a reliable visual field was defined as having <30% false-positive errors, <30% false-negative errors, and <20% fixation losses. Patients with retinal disease likely to affect visual field measurements, intraocular surgery within the preceding 6 months, or active ocular infection were excluded. The participant was considered the unit of analysis. When both eyes fulfilled the eligibility criteria, the eye with greater glaucoma severity was selected based on the HFA visual field mean deviation (MD). Glaucoma severity was classified according to the Hodapp–Parrish–Anderson criteria as mild (MD ≥ −6 dB), moderate (MD < −6 to ≥ −12 dB), or severe (MD < −12 dB).

### 2.2. Sample Size

The required sample size was 57 participants, calculated using Power Analysis and Sample Size (PASS) software (version 11.0.7; NCSS, LLC, Kaysville, UT, USA), based on the Bland–Altman method using previously published agreement data between MRF and HFA [17]. The calculation assumed a statistical power of 80% and a significance level of 5%. Based on the study by Kong et al., the expected mean difference between HFA and MRF was 3.1 dB for MD, with a standard deviation of the differences of 1.2 dB and a maximum allowable difference of 11.6 dB. For PSD/PD, the expected mean difference was 2.0 dB, with a maximum allowable difference of 9.7 dB. As the previous study did not report Bland–Altman limits of agreement for VFI/VC, values reported by Schulz et al. were used, with an expected mean difference of 2% and a maximum allowable difference of 30%.

### 2.3. Visual Function Assessment

At the study visit, demographic and clinical information, including age, sex, ethnicity, glaucoma type, duration of glaucoma, and previous ocular surgery, was recorded using a standardized data collection form. All participants underwent visual acuity assessment using a Snellen chart, slit-lamp anterior segment examination, and undilated fundus examination using a 90-diopter condensing lens. Goldmann applanation tonometry (GAT) was not performed before visual field testing to avoid any potential influence of tonometry-related corneal or ocular discomfort on perimetric performance. A single GAT measurement was obtained after completion of both visual field tests.

All participants underwent HFA before MRF. The sequence was fixed according to the study protocol and was not randomized. Standard automated perimetry was performed using the Humphrey Field Analyzer model 750i (Carl Zeiss Meditec AG, Jena, Germany) with the Swedish Interactive Thresholding Algorithm (SITA) 24-2 Standard strategy. HFA 24-2 SITA-Standard was selected because it was the established standard automated perimetry strategy routinely used in our clinic during the study period and was also the comparator used in the earlier MRF validation studies on which the study design was based. Testing was performed according to the manufacturer’s standard instructions. Participants were instructed to maintain fixation on the central fixation target and to respond whenever a stimulus was perceived, while avoiding attempts to search for stimuli. Appropriate near correction was provided when required. The fellow eye was occluded during testing, and the participant’s head and chin were positioned comfortably on the instrument’s support. The following HFA parameters were recorded: fixation losses, false-positive and false-negative rates, mean deviation (MD), pattern standard deviation (PSD), visual field index (VFI), and test duration.

Following HFA testing, participants were allowed to rest for at least 5–10 min before undergoing MRF testing. A longer rest period (>30 min) was provided when participants reported significant fatigue. MRF testing was performed using an 11-inch iPad Pro running iOS 14.0 in a quiet room with dim background illumination. The tablet brightness was automatically set to maximum by the application. Participants wore their appropriate near-vision correction when required. The MRF examination was performed monocularly, with the fellow eye occluded, as shown in Figure 1. The application used the device’s front-facing camera to guide positioning and maintain a viewing distance of approximately 33 cm. Participants were instructed to maintain fixation on the central target and to avoid head movement during testing. Because participants were experienced HFA users but had not previously undergone MRF perimetry, a brief practice test was performed before the full MRF examination to familiarize participants with the tablet interface, fixation target and response procedure.

MRF uses a black background with a central fixation target and visual stimuli presented at predefined locations. At a viewing distance of 33 cm, the iPad provides a visual field of approximately 30° × 24°. The 24-2 testing strategy consisted of 66 test locations. Because of the limited physical screen size, peripheral locations were assessed by shifting fixation toward each of the four corners of the display, thereby extending the tested field as shown in Figure 1. The application initially identified the physiological blind spot before performing threshold testing. Voice prompts were provided throughout the examination to guide the participant and encourage fixation. The investigator monitored the participant’s head position and paused the test when substantial movement occurred.

An example of the MRF report is shown in Figure 2. MRF reports pattern deviation (PD) and visual capacity (VC), whereas HFA reports pattern standard deviation (PSD) and visual field index (VFI). Because these are device-specific indices derived using different algorithms and normative databases, PD and PSD, and VC and VFI, were treated as analogous parameters for comparative analysis rather than mathematically equivalent measures. Standardized manufacturer-defined reliability thresholds for MRF have not been established; hence, a study-specific operational definition was used: an MRF examination was considered unreliable when fixation loss reached 100% or when either false-positive or false-negative responses exceeded 50%. These thresholds were defined a priori for participant exclusion and should not be interpreted as equivalent to the conventional HFA reliability criteria.

To assess test–retest reliability, participants underwent a second MRF examination 4 weeks after the initial examination. The same full-field 24-2 thresholding strategy was used for both examinations. Following the MRF examination, participants completed a brief questionnaire assessing their experience with the test, including its acceptability and feasibility. The questionnaire evaluated participants’ perceptions of the testing procedure and was completed after the initial MRF examination.

### 2.4. Statistical Analysis

Data were analyzed using IBM SPSS Statistics version 26.0 (IBM Corp., Armonk, NY, USA). Data distribution was assessed using histograms, skewness, and kurtosis. Paired-samples *t*-tests were used to compare MD, PSD/PD, VFI/VC, and test duration between HFA and MRF. Agreement between the two perimetric methods was evaluated using intraclass correlation coefficients (ICC) and Bland–Altman analysis. ICC estimates were interpreted according to commonly accepted thresholds: <0.50, poor reliability; 0.50–0.75, moderate reliability; 0.75–0.90, good reliability; and >0.90, excellent reliability [23].

Bland–Altman analysis was performed for MD, PSD/PD, and VFI/VC. The bias was calculated as the mean difference between HFA and MRF measurements, with the corresponding 95% limits of agreement (LoA) calculated as mean difference ± 1.96 × SD of the differences. Bland–Altman plots displayed the mean of the two methods on the x-axis and the difference between HFA and MRF measurements on the y-axis. The presence of proportional bias was assessed using linear regression analysis, with statistical significance defined as *p* < 0.05. Test–retest reliability of MRF measurements was assessed using a two-way mixed-effects, single-measure intraclass correlation coefficients (ICC) with absolute agreement with 95% confidence intervals. Pearson correlation coefficients were additionally calculated to assess the correlation between test and retest measurements. Correlation coefficients of 0.50–1.00 were considered strong, 0.30–0.49 moderate, and <0.30 weak. A two-sided *p* < 0.05 was considered statistically significant. Generative AI (GenAI) tools were used to assist with graph enhancement.

## 3. Results

Seventy patients were recruited, of whom 64 completed reliable HFA and MRF examinations and were included in the analysis. Six participants were excluded because of unreliable perimetry: two had unreliable HFA results and four had 100% fixation loss on MRF. A prolonged rest period (>30 min) was permitted in two participants who reported significant fatigue after the HFA. Forty-seven participants subsequently completed repeat MRF testing at 4 weeks; the remaining participants were unable to return within the prespecified interval because of movement restrictions during the study period. The participant flow is shown in Figure 3.

The mean age of the 64 participants was 60.8 ± 12.9 years (range, 19–79 years), with 35 (54.7%) being male. Primary glaucoma accounted for 55 (85.9%) participants. The secondary glaucoma group comprised 4 cases of pseudoexfoliative glaucoma, 2 cases of uveitic glaucoma, 2 cases of traumatic glaucoma, and 1 case of neovascular glaucoma. Based on HFA MD, 25 eyes had mild, 24 moderate, and 15 severe glaucoma. The median baseline IOP was 15.0 mmHg (IQR, 14.0–17.8 mmHg). Baseline demographic and clinical characteristics of the overall cohort (*n* = 64) and the subgroup who completed repeat MRF testing (*n* = 47) are presented descriptively in Table 1.

MRF demonstrated excellent test–retest reliability across the three global indices, with ICCs ranging from 0.992 to 0.993. The mean differences between baseline and week 4 were small for MD (−0.14 dB), PD (+0.20 dB), and VC (+0.01%). The corresponding 95% limits of agreement were −2.00 to 1.72 dB for MD, −0.89 to 1.29 dB for PD, and −6.06% to 6.08% for VC, with coefficients of repeatability of 1.86 dB, 1.09 dB, and 6.07%, respectively (Table 2). Despite the excellent ICC values, ICC reflects relative reliability within the cohort rather than individual measurement precision and observed absolute variability. The coefficient of repeatability (CoR) of 1.86 dB indicates that changes in MD exceeding 1.86 dB between repeated measurements within the same eye are more likely to support true disease progression.

The numerical reliability indices recorded during MRF and HFA testing were similar in this cohort. Mean fixation loss was 7.64 ± 12.66% with MRF and 6.45 ± 7.37% with HFA (*p* = 0.435), while mean false-positive and false-negative rates with MRF were 1.55 ± 4.61% and 4.33 ± 8.40%, respectively, with no statistically significant differences compared with HFA (*p* = 0.295 and *p* = 0.172, respectively). Because the two platforms used different reliability thresholds to define an unreliable examination, these findings represent a comparison of numerical reliability indices rather than validation of equivalent clinical reliability criteria. Among the 47 participants who underwent repeat MRF testing at 4 weeks, test–retest reproducibility was excellent. ICCs were 0.993 (95% CI, 0.987–0.996) for MD, 0.992 (95% CI, 0.985–0.996) for PD, and 0.992 (95% CI, 0.985–0.995) for VC (all *p* < 0.001). These findings indicate high inter-session reproducibility of MRF measurements.

Despite high consistency between the two instruments, systematic differences were observed in some global indices. MRF produced significantly less negative MD values than HFA (−6.27 ± 5.55 vs. −8.32 ± 5.47 dB; mean difference, −2.05 dB; *p* < 0.001) and significantly higher PD values (7.48 ± 3.26 vs. 6.33 ± 4.11 dB; mean difference, −1.15 dB; *p* < 0.001). In contrast, VC was similar between MRF and HFA (82.19 ± 17.19% vs. 82.45 ± 16.36%; mean difference, 0.26%; *p* = 0.808). Agreement between the two instruments was excellent for MD (ICC, 0.949; 95% CI, 0.916–0.969) and VFI/VC (ICC, 0.930; 95% CI, 0.884–0.957), and good for PSD/PD (ICC, 0.880; 95% CI, 0.802–0.927) (Table 3). Bland–Altman analysis demonstrated a mean difference of −2.05 dB for MD, −1.15 dB for PSD/PD, and 0.26% for VFI/VC. The corresponding 95% limits of agreement were −6.81 to 2.71 dB, −5.91 to 3.61 dB, and −16.59 to 17.12%, respectively. Proportional bias was not detected for MD or VFI/VC, whereas PSD/PD demonstrated significant proportional bias (β = 0.254, *p* = 0.003) (Figure 4).

The degree of agreement varied according to glaucoma severity and the visual-field parameter examined (Table 4). For MD, the mean difference increased with glaucoma severity, from −1.22 dB in mild glaucoma to −2.53 dB in moderate and −2.69 dB in severe glaucoma. Although proportional bias was detected in the moderate and severe groups, the mean differences remained relatively small. For PSD/PD, the smallest mean difference was observed in severe glaucoma (0.25 dB), with limits of agreement of −3.24 to 3.74 dB. The moderate group demonstrated the widest limits of agreement (−6.36 to 4.50 dB). No significant proportional bias was detected within any severity group. For VFI/VC, the mild glaucoma group demonstrated the narrowest limits of agreement (−7.03 to 8.49%), whereas agreement was substantially wider in moderate and severe glaucoma (−18.86 to 20.54% and −24.28 to 21.38%, respectively). Proportional bias was observed only in the moderate glaucoma group (*p* = 0.019). In these exploratory subgroup analyses, MRF showed narrower limits of agreement for MD and VC in mild glaucoma, whereas PD showed relatively narrower limits of agreement in severe glaucoma. These observations should be interpreted cautiously given the small and unequal subgroup sizes.

MRF was significantly faster than HFA, with a mean test duration of 4.22 ± 0.81 versus 5.46 ± 1.33 min, representing a mean reduction of 1.25 min (95% CI, 0.97–1.53; *p* < 0.001). The reduction in test duration remained significant across mild, moderate, and severe glaucoma (all *p* < 0.001) as shown in Figure 5. The greatest time saving was observed in severe glaucoma, with a mean reduction of 1.62 min. MRF was also highly acceptable to participants as shown in Table 5. All 64 participants reported that the test was easy and comfortable, and none reported feeling stressed during testing. Sixty-three participants (98.4%) indicated that they would be willing to repeat the test, and 63 (98.4%) preferred MRF over HFA. Only one participant reported no preference between the two methods. Although 63 participants did not own an iPad, this did not prevent successful completion of the test in the clinic.

## 4. Discussion

Portable digital technology has increasingly been incorporated into ophthalmic assessment, including visual acuity, contrast sensitivity and retinal sensitivity testing [24,25,26]. The development of tablet-based perimetry represents a further extension of this technology, with the potential to make visual field assessment more portable, accessible and frequent. This is particularly relevant in glaucoma, where repeated visual field testing is essential for detecting functional progression but is constrained by clinic capacity, equipment availability and patient burden. Previous studies have demonstrated the feasibility and clinical performance of MRF compared with HFA, but these studies have predominantly involved Australian, European or Indian populations [17,18,19,20]. To our knowledge, this is the first study to evaluate the agreement, repeatability, test duration and patient acceptability of MRF compared with HFA 24-2 SITA Standard in a multi-ethnic Malaysian glaucoma cohort, including Malay, Chinese and Indian participants.

### 4.1. Reliability and Repeatability of MRF

The numerical reliability indices recorded during MRF including fixation loss, false-positive and false-negative were generally low and did not differ significantly from those observed with HFA in our cohort. This finding is consistent with previous MRF studies, in which false-positive and false-negative rates were generally within the range expected for reliable visual fields [18,19,20]. However, interpretation of MRF reliability indices requires some caution because standardized manufacturer-defined reliability thresholds equivalent to those routinely used for HFA are not well established. Comparable differences in fixation losses, false-positive rates, and false-negative rates should not be interpreted as demonstrating equivalent clinical reliability between MRF and HFA. In our study, only five participants had false-positive rates > 25% and two had false-negative rates > 25%, suggesting that excessive response bias was uncommon.

Fixation loss was more variable with MRF, with a mean of 7.64 ± 12.66%. This may partly reflect the unfamiliarity of participants with tablet-based testing and the absence of a forehead or chin rest to stabilize the head. The finding is also consistent with the practical challenges reported in home-based tablet perimetry, where fixation control and test positioning are less constrained than with conventional bowl perimetry [14,18,19,20]. The failure of MRF to detect the blind spot in 23.4% of participants at the initial visit and 29.8% during repeat testing further highlights the importance of fixation monitoring when interpreting MRF results. These proportions were somewhat higher than those reported by Schulz et al. and Kumar et al., who reported failure of blind-spot detection in approximately 18% and 14% of participants, respectively [18,20].

The higher frequency of missed blind-spot detection in our study may reflect a learning effect. All participants were experienced HFA users but were naïve to MRF, and almost all had no previous experience using an iPad for visual field testing. Familiarization with the tablet interface, positioning and fixation target may therefore be particularly important during the first examination. Future studies evaluating MRF in unsupervised settings should consider whether repeated familiarization tests or improved eye/head tracking could improve fixation reliability.

Despite these limitations, MRF demonstrated excellent test–retest reproducibility over 4 weeks, with ICCs of approximately 0.99 for MD, PD and VC. This finding is consistent with previous studies demonstrating good short- and medium-term repeatability of MRF [18,19,20,27]. The high ICCs observed in our study support the ability of MRF to provide consistent measurements when the test is repeated under similar conditions.

Importantly, however, repeatability and agreement are different properties. A device may produce highly reproducible measurements without producing measurements that are numerically interchangeable with another perimeter. This distinction is particularly relevant to our subsequent comparison with HFA.

### 4.2. Agreement Between MRF and HFA

MRF produced significantly less negative MD values and significantly higher PSD/PD values than HFA, whereas VFI/VC did not differ significantly between the two methods. The direction of these differences is consistent with previous MRF-HFA comparisons. Kumar et al. reported a significantly less negative MD and higher PSD/PD with MRF, while Kong et al. also observed differences in MD and PSD/PD, particularly in patients with moderate-to-severe glaucoma [17,20].

These systematic differences are likely multifactorial. MRF and HFA differ in stimulus presentation, threshold estimation algorithms, stimulus size and luminance characteristics, as well as their normative databases. MRF estimates retinal sensitivity using a Bayesian approach and a limited number of discrete stimulus levels, whereas HFA SITA uses an adaptive pointwise thresholding strategy [28,29,30]. Differences in the normative database and in the calculation of global indices may therefore contribute to differences in the absolute values reported by the two instruments. More recent comparative work has similarly demonstrated that apparently similar global indices can coexist with clinically meaningful point-wise differences between tablet-based perimetry and HFA, potentially related to differences in normative databases and device-specific algorithms [13].

The absence of a significant difference in VFI/VC between MRF and HFA is encouraging, as VFI/VC is expressed as a percentage relative to age-corrected normal sensitivity and is less directly dependent on the absolute sensitivity scale. Previous MRF studies have likewise reported relatively close agreement in VFI/VC between MRF and HFA [17,18,20]. Nevertheless, VFI/VC should not be interpreted as completely device-independent. Differences in the underlying sensitivity measurements and normative datasets can still influence the final VFI/VC calculation [13].

Although ICC demonstrated good-to-excellent agreement between MRF and HFA for MD, PSD/PD and VFI/VC, Bland–Altman analysis provided a more clinically informative assessment of interchangeability. The mean differences were −2.05 dB for MD, −1.15 dB for PSD/PD and 0.26% for VFI/VC. The 95% limits of agreement were −6.81 to 2.71 dB for MD, −5.91 to 3.61 dB for PSD/PD and −16.59% to 17.12% for VFI/VC. Proportional bias was not identified for MD or VFI/VC but was present for PSD/PD.

These findings emphasize an important distinction: high correlation or ICC does not necessarily imply that two methods can be used interchangeably for individual clinical decisions. Bland–Altman analysis is particularly useful in this context because it evaluates the magnitude and distribution of disagreement across the measurement range rather than simply assessing whether the two measurements vary together [29]. In our study, the relatively wide limits of agreement indicate that although MRF can provide broadly comparable information at the group level, an individual patient’s MRF global indices should not simply be substituted for HFA values.

This interpretation is also supported by more recent comparative data. Kang et al. found that although average MD and PSD/PD measurements from MRF and HFA were generally similar, substantial differences remained at individual visual field locations, with systematic regional differences in sensitivity [13]. Thus, our findings add to the growing evidence that tablet perimetry should be viewed as a complementary perimetric platform rather than a direct replacement for conventional SAP.

### 4.3. Influence of Glaucoma Severity

An important finding of our study was that agreement varied according to glaucoma severity. This aspect has received relatively little attention in previous MRF-HFA studies. For MD, the magnitude of the difference increased with glaucoma severity, from −1.22 dB in mild glaucoma to −2.53 dB in moderate and −2.69 dB in severe glaucoma. Although these mean differences remained within our prespecified agreement threshold, proportional bias was present in the moderate and severe groups. This suggests that the difference between MRF and HFA may become more pronounced as the visual field becomes more depressed. One possible explanation is that device-specific differences in threshold estimation and normative databases become increasingly important at lower sensitivity levels. The limited discrete sensitivity steps used by MRF may also result in different threshold estimates when sensitivity is substantially reduced. In addition, the dynamic range and stimulus characteristics of the two devices differ [28,29].

For PSD/PD, the pattern was different. The severe glaucoma group demonstrated the smallest mean difference and the narrowest limits of agreement, with no evidence of proportional bias. This may reflect the nature of PSD/PD as a measure of localized variability within the visual field rather than an absolute measure of overall sensitivity. However, this finding should be interpreted cautiously because the severe glaucoma subgroup contained only 15 participants.

VFI/VC showed the clearest severity-dependent variability. Agreement was best in mild glaucoma, with limits of agreement of approximately −7.0% to +8.5%, whereas the limits widened considerably in moderate and severe glaucoma. Proportional bias was also observed in the moderate group. Thus, while VFI/VC showed minimal systematic difference between MRF and HFA overall, the variability between the two methods increased substantially in eyes with more advanced field loss.

Taken together, these findings suggest that there is no single measure of “agreement” that applies equally across all stages of glaucoma. MRF MD and VFI/VC appear most comparable to HFA in mild glaucoma, whereas PSD/PD showed relatively closer agreement in severe disease. However, the subgroup analyses were exploratory and should not be interpreted as establishing stage-specific interchangeability. Larger studies with balanced numbers of mild, moderate and severe cases are required to confirm these observations.

### 4.4. Test Duration and Patient Acceptability

A major practical advantage of MRF in our study was its shorter testing time. MRF required approximately 4.22 min compared with 5.46 min for HFA, representing a mean reduction of 1.25 min. This finding is consistent with previous MRF studies reporting shorter testing times than HFA [18,19,20]. The reduction in test duration was observed across all glaucoma severity groups and was greatest in severe glaucoma. The shorter duration is likely related to differences in thresholding strategy. HFA SITA Standard uses an adaptive pointwise thresholding algorithm to estimate sensitivity at multiple locations, whereas MRF uses a Bayesian approach to estimate thresholds from a defined response matrix [15,16]. Previous work has demonstrated that response time varies across the visual field and is influenced by both stimulus characteristics and thresholding strategy [30].

The choice of HFA SITA-Standard also has implications for interpretation of test duration. SITA-Faster is a more rapid HFA strategy; however, differences in visual-field measurements may occur when switching between SITA-Standard and SITA-Faster, particularly in moderate and advanced glaucoma [31]. Pham et al. reported higher MD values after transition to SITA-Faster in moderate and advanced glaucoma, highlighting that test duration should be interpreted within the context of the specific HFA strategy used [31].

The shorter test duration may have practical clinical importance. Reducing the burden of perimetry could improve patient tolerance and potentially permit more frequent testing, particularly in busy glaucoma clinics. More frequent testing is clinically relevant because detection of visual field progression depends not only on the accuracy of individual tests but also on the number and timing of measurements obtained over time. The ability to perform more frequent assessments is one of the major proposed advantages of portable perimetry.

Patient acceptability in our study was particularly encouraging. All participants considered MRF easy and comfortable, none reported feeling stressed during the test, and 98.4% were willing to repeat the examination. Similarly, 98.4% preferred MRF over HFA. The repeated voice instructions and the absence of the relatively restrictive bowl and head-positioning requirements of HFA may have contributed to this positive experience.

However, these findings should be interpreted as acceptability rather than evidence of clinical superiority. The questionnaire was completed immediately after the MRF examination, and participants were already familiar with HFA but were experiencing MRF for the first time. Future studies should use validated patient-reported outcome measures and compare both tests using a randomized test order to minimize potential order and novelty effects.

### 4.5. Implications for Home and Community-Based Testing

The portability and shorter testing time of MRF raise the possibility of extending visual field assessment beyond the conventional glaucoma clinic. This is particularly relevant in health systems where access to standard automated perimetry is limited by equipment availability, cost, geographic distance or clinic workload.

The potential for home monitoring is supported by studies using MRF and other tablet-based perimeters. Jones et al. demonstrated that patients with glaucoma could perform repeated tablet-based visual field testing at home over 6 months, with high adherence and good agreement with clinic-based measurements [14]. Prea et al. similarly demonstrated high uptake and persistence of weekly home visual field monitoring using an iPad-based MRF system, although information-technology and motivation-related factors remained barriers to sustained participation [19]. More recently, Kong et al. evaluated unsupervised MRF online perimetry performed at home and reported strong agreement for MD with clinic-based HFA SITA-Faster and good MRF test–retest reproducibility, while agreement for pattern-based indices was more modest. These findings provide encouraging evidence for home-based MRF but also reinforce that device-specific global indices should not be assumed to be interchangeable across platforms [32].

However, our study should not be interpreted as validating MRF for unsupervised home monitoring, because all examinations were performed in a controlled clinical environment under investigator supervision. This distinction is important. A systematic review of home-based perimetry found promising results but highlighted that most studies were conducted in controlled settings and highly selected populations, with uncertainty remaining regarding real-world diagnostic performance and clinical value [21].

Our findings therefore support MRF as a potentially useful complement to, rather than replacement for, conventional HFA. In the clinic, MRF could potentially be used for rapid visual field assessment, particularly in settings where access to HFA is limited. Outside the clinic, it may eventually have a role in remote or community-based monitoring, but this would require appropriate validation of unsupervised testing, fixation control, viewing distance, ambient lighting, data transfer and patient adherence.

### 4.6. Limitations

Several limitations should be considered. First, all MRF examinations were performed under investigator supervision in a controlled clinical environment. This may have produced better performance than would be expected during unsupervised home testing. Although standardized instructions were provided, the influence of supervision cannot be excluded.

Second, the study population was deliberately restricted to patients with established glaucoma who were experienced HFA users. The inclusion of heterogeneous secondary glaucoma mechanisms may have introduced additional variability in visual-field characteristics and limits generalizability to individual glaucoma subtypes. Pre-perimetric glaucoma and healthy controls were not included. Consequently, our findings primarily address agreement and repeatability in established glaucoma and cannot establish the diagnostic accuracy of MRF for glaucoma screening or early disease detection. Furthermore, the glaucoma severity groups were relatively small and unequal, particularly the severe group. The severity-specific Bland–Altman findings should therefore be considered exploratory rather than definitive. When both eyes were eligible, the eye with greater glaucoma severity was selected for analysis to avoid inter-eye clustering. This approach may have resulted in preferential representation of more advanced disease and may limit generalizability to the broader glaucoma population.

Although a brief practice test for MRF perimetry was provided, a residual learning effect cannot be excluded because participants were experienced HFA users but MRF-naïve. In addition, a further limitation is the fixed testing sequence, with HFA always performed before MRF. Although participants were allowed to rest for at least 5–10 min and a longer rest period was provided when substantial fatigue was reported, residual fatigue or attention effects may have influenced the subsequent MRF examination. Conversely, participants were HFA-experienced but MRF-naïve, introducing a potential learning or novelty effect. The shorter MRF test duration and high preference should therefore be interpreted in the context of this non-randomized sequence. Future comparative studies should randomize test order and use standardized familiarization procedures.

Moreover, our study evaluated global indices rather than detailed point-by-point agreement across the visual field. This is relevant because recent comparative studies have demonstrated that portable tablet perimeters may show systematic differences at individual test locations despite relatively similar global MD values [13]. Future work should therefore investigate point-wise sensitivity, scotoma detection and progression rates, particularly in early glaucoma.

As the 17 participants who did not complete repeat MRF testing were unable to return due to movement restrictions during the study period, the repeatability findings were based solely on the 47 participants who completed follow-up. Since baseline characteristics were not directly compared between participants who completed repeat testing and those who did not, potential attrition bias cannot be excluded.

### 4.7. Future Directions

Future studies should evaluate MRF in a larger and more diverse population, including glaucoma suspects, pre-perimetric glaucoma and healthy controls, and should assess diagnostic accuracy as well as longitudinal progression. Randomized test order and standardized familiarization procedures would reduce potential learning and fatigue effects. The next step should also be evaluation in real-world home and community settings. This should include assessment of adherence, test failure rates, viewing-distance control, fixation monitoring, ambient lighting, data security and the practical requirements for storing and transmitting repeated visual field results. Cost-effectiveness is another important consideration before widespread implementation.

Importantly, the objective of home or portable perimetry should not simply be to reproduce an HFA examination outside the clinic. Its greater potential may lie in enabling more frequent longitudinal measurements. Weekly or otherwise more frequent home testing could theoretically provide a denser time series of visual field measurements and improve the detection of clinically meaningful progression, although this remains to be demonstrated in prospective clinical studies [14,19,21].

## 5. Conclusions

In this Malaysian glaucoma cohort, MRF demonstrated excellent test–retest reproducibility and good-to-excellent overall agreement with HFA for MD, PSD/PD and VFI/VC. However, systematic differences and relatively wide limits of agreement, particularly in moderate and severe glaucoma, indicate that MRF should not be considered interchangeable with HFA for individual clinical decision-making. MRF was approximately 1.25 min faster than HFA and was highly acceptable to participants, supporting its potential role as a portable and patient-friendly adjunct to conventional perimetry. Our findings suggest that the most appropriate role for MRF at present is not to replace HFA, but to extend access to visual field testing—particularly where portability, shorter testing time and the possibility of more frequent assessment are advantageous. Future studies utilizing a randomized testing sequence and a validated questionnaire are essential to further evaluate patient acceptability. Further validation in home and community settings is required before MRF can be recommended for unsupervised monitoring or population-based glaucoma screening.

## Figures and Tables

**Figure 1:**
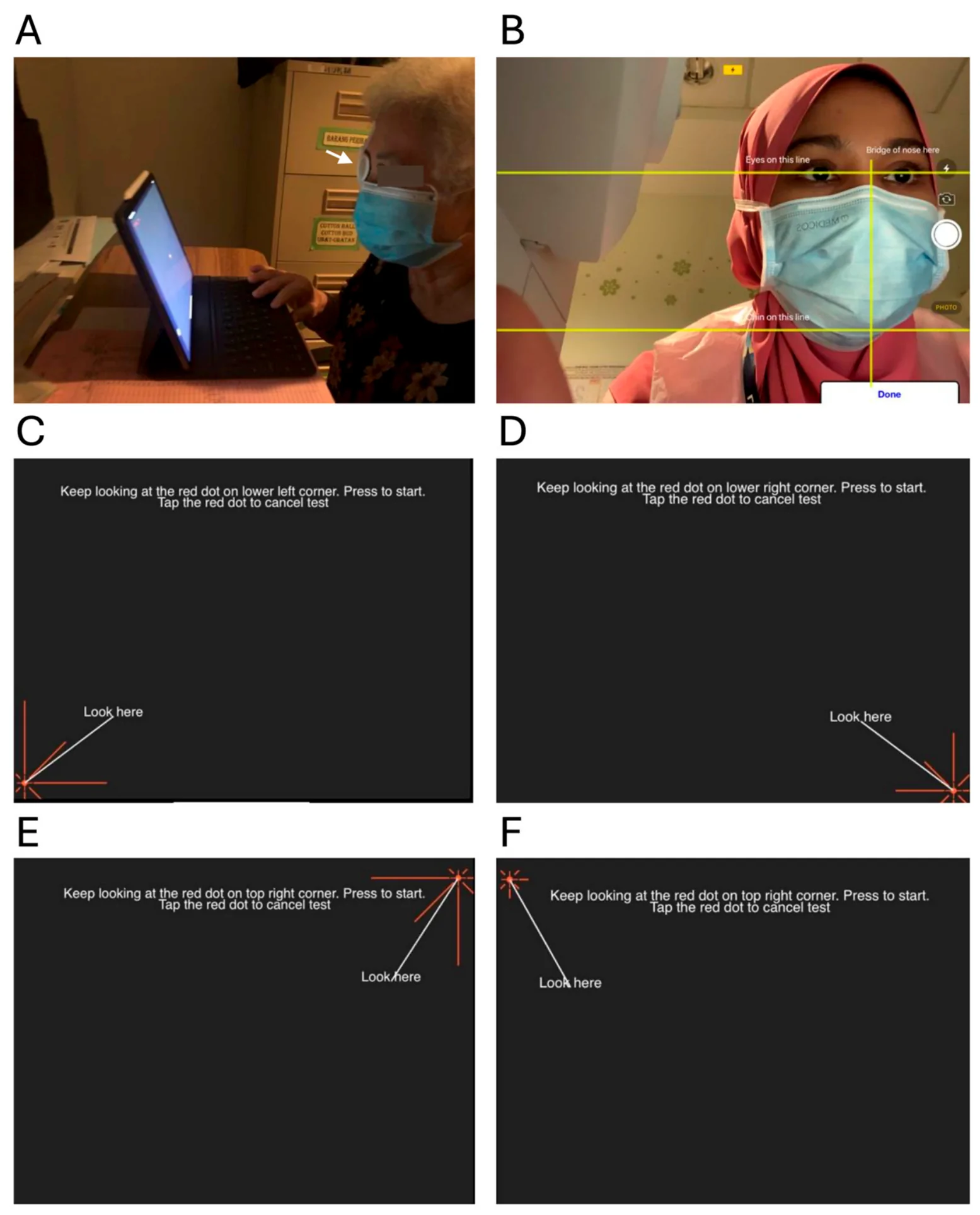
(**A**) A participant was sitting comfortably and performing the Melbourne Rapid Fields (MRF) test. One eye was occluded (white arrow) when testing an eye. The photograph is published with the patient’s written informed consent. (**B**) Screenshot from the Melbourne Rapid Fields (MRF) application camera displaying how the eye distance was confirmed to be 33 cm from the bridge of the nose to the iPad. (**C**–**F**) Due to the small screen size of approximately 15 × 12°, the test requires the subject to fixate in the 4 corners of the screen (**A**–**D**) at different times to gain eccentricities out to 30 degrees.

**Figure 2:**
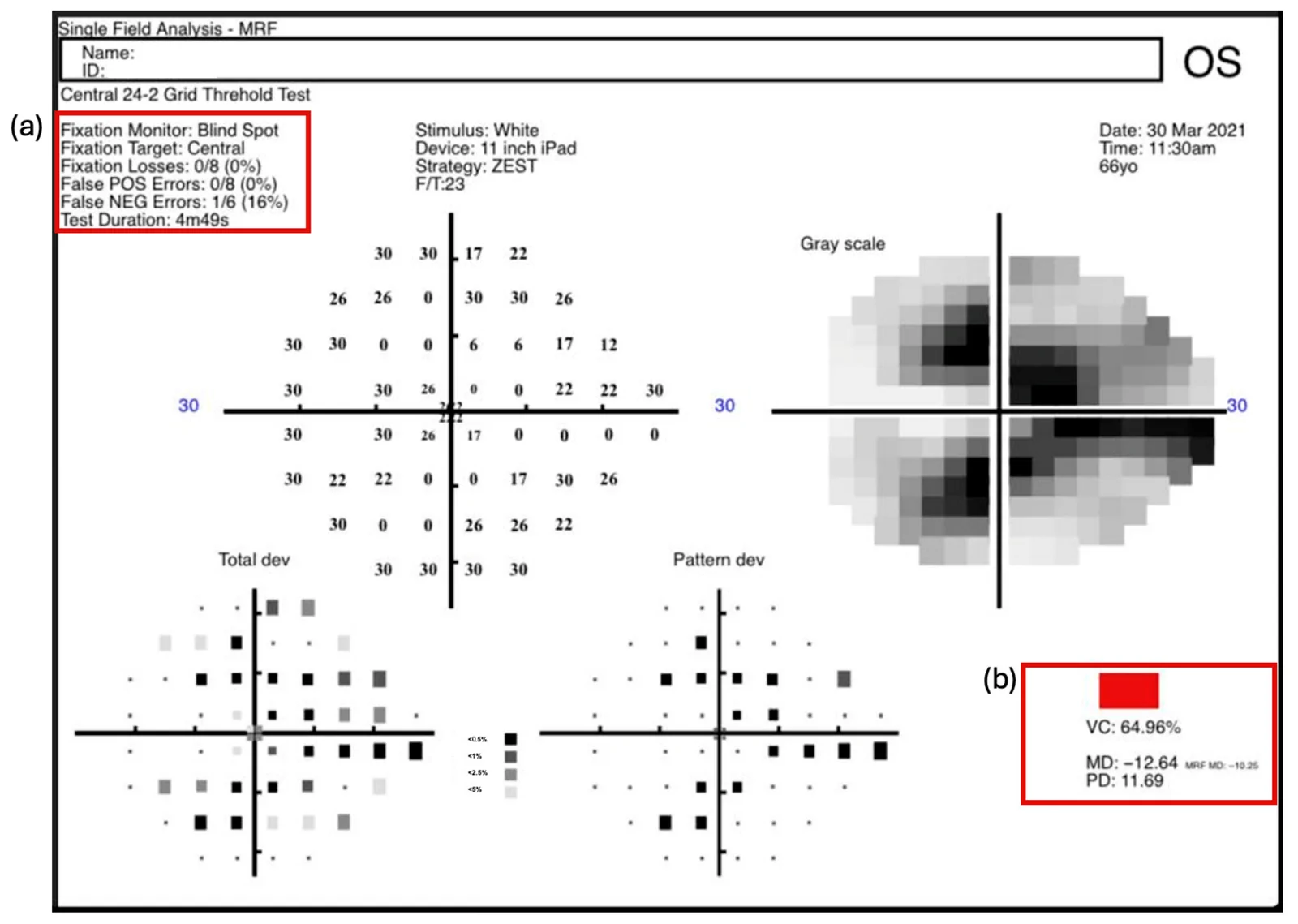
Example of Melbourne Rapid Fields (MRF) full field threshold 24-2 result test. Results included: (**a**) reliability indices value (fixation losses, false positive, false negative) and test duration, and (**b**) global indices (mean deviation [MD], pattern deviation [PD] and visual capacity [VC] percentage), highlighted by the red boxes. Abbreviations: MRF, Melbourne Rapid Fields; OS, ocular sinister (left eye); POS, positive; NEG, negative; ZEST, Zippy Estimation by Sequential Testing; dev, deviation; VC, visual capacity; MD, mean deviation; PD, pattern deviation.

**Figure 3:**
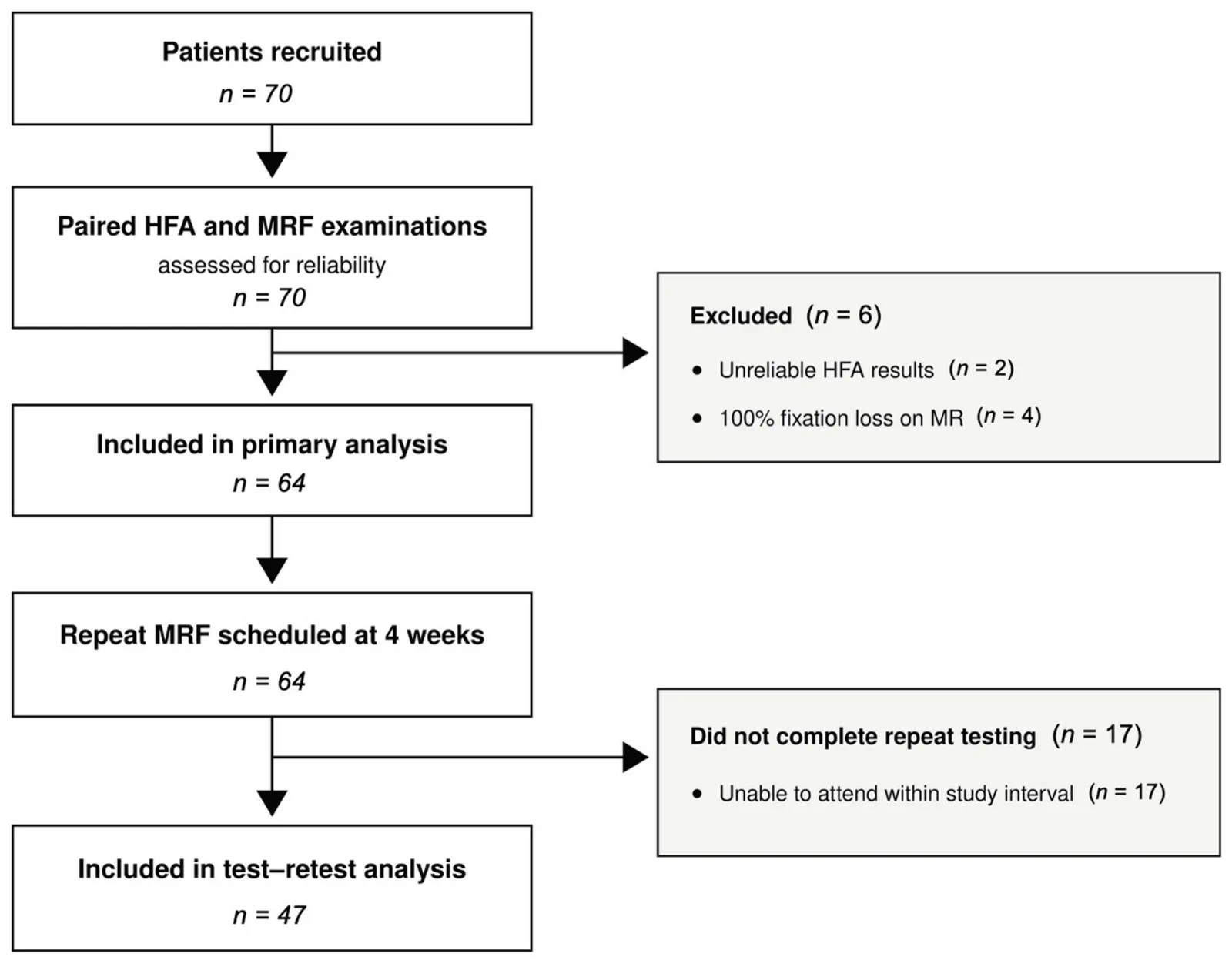
Participant flow diagram.

**Figure 4:**
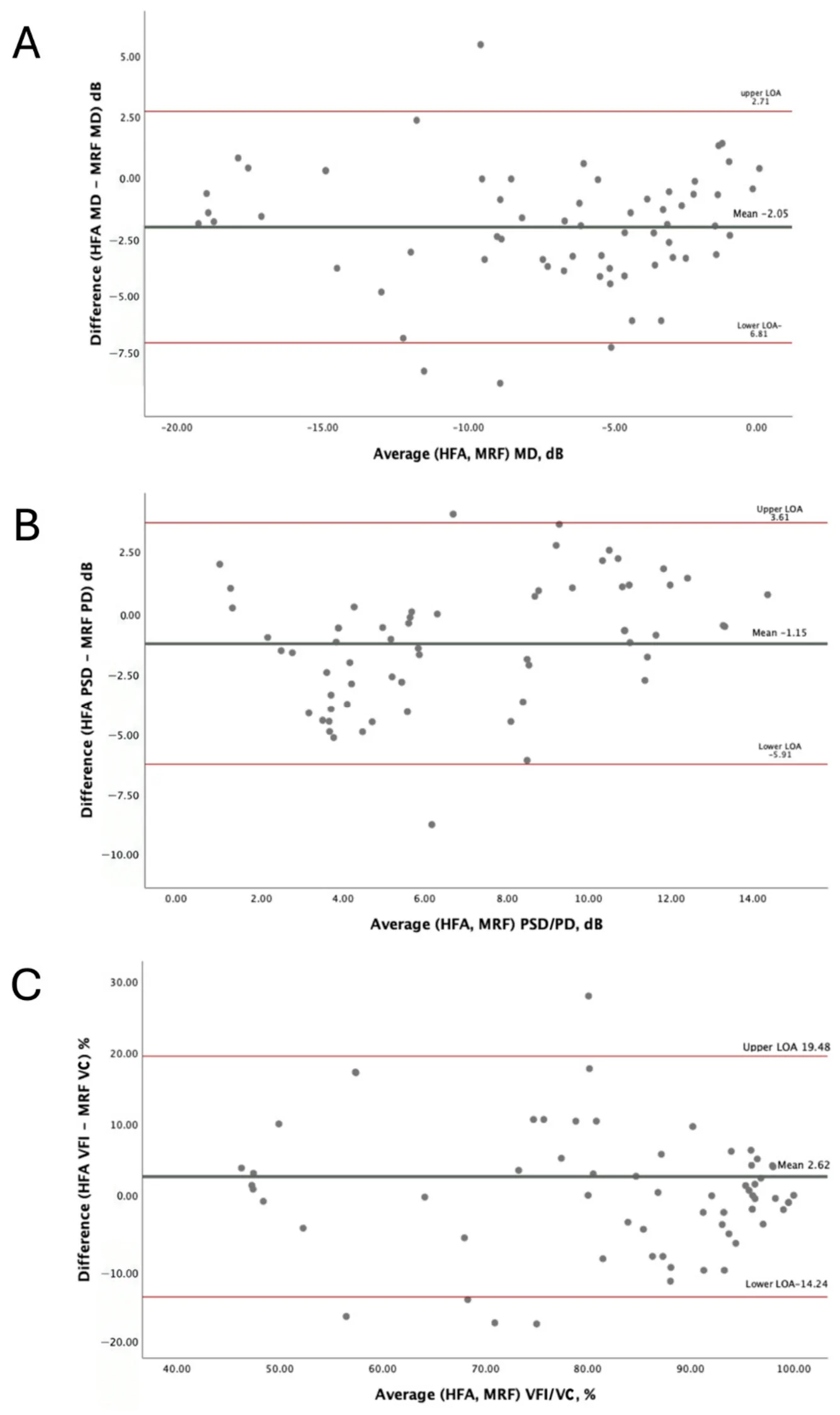
Bland–Altman plots comparing the Humphrey Field Analyzer (HFA) and Melbourne Rapid Fields (MRF) test for (**A**) Mean Deviation (MD) agreement, (**B**) Pattern Standard Deviation (PSD) agreement, and (**C**) Visual Field Index (VFI) agreement. Abbreviations: HFA, Humphrey Field Analyzer; MRF, Melbourne Rapid Fields; dB, decibels; MD, mean deviation; PSD, pattern standard deviation; PD, pattern deviation; VFI, visual field index; VC, visual capacity; LOA, limits of agreement.

**Figure 5:**
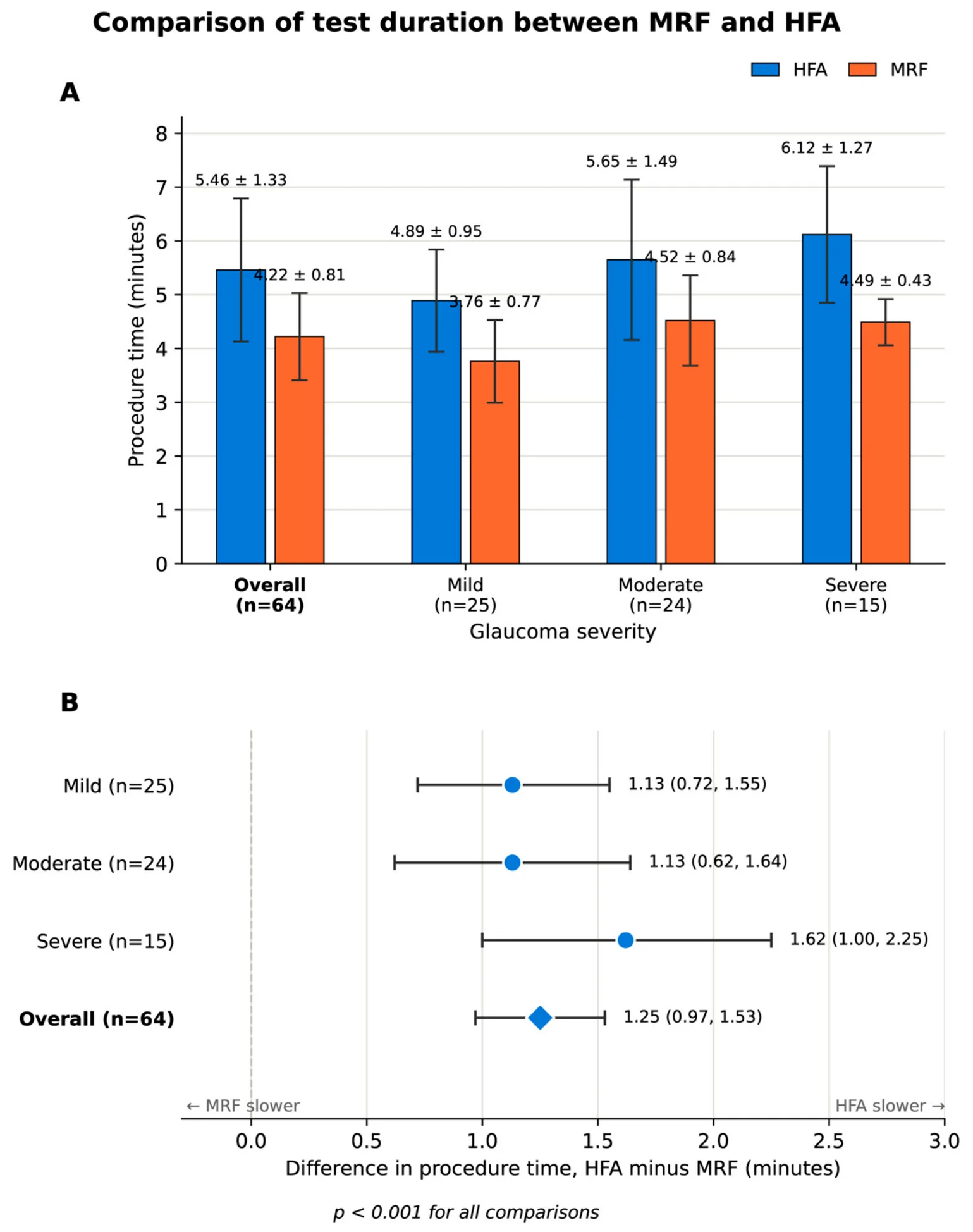
MRF vs. HFA test duration by glaucoma severity. (**A**) Comparison of test duration, (**B**) difference between test duration.

**Table 1:** Demographic data of the study population and test–retest reliability at the study visit.

Variables		Study Visit (*n* = 64)	Test–Retest Reliability (*n* = 47)
Age in years			
Mean ± SD Range		60.77 ± 12.9 19–79	60.26 ± 14.02 19–79
Gender, *n* (%)	Male Female	35 (54.7) 29 (45.3)	26 (55.3) 21 (44.7)
Ethnicity, *n* (%)	Malay Chinese Indian	30 (46.9) 24 (37.5) 10 (15.6)	23 (48.9) 17 (36.2) 7 (14.9)
Type of glaucoma, *n* (%)	Primary glaucoma Secondary glaucoma	55 (85.9) 9 (14.1)	40 (85.1) 7 (14.9)
IOP, mmHg, median (IQR)		15.0 (14.0, 17.8)	16.0 (14.8, 18.8)
Duration of glaucoma, years, median (IQR)		7 (3, 11)	6.6 (2, 11)
CDR, mean ± SD		0.74 ± 0.10	0.74 ± 0.10
Snellen visual acuity, logMAR, mean ± SD		0.14 ± 0.09	0.16 ± 0.09
Previous ocular surgery, *n* (%)	Yes No	22 (34.4) 42 (65.6)	17 (36.2) 30 (63.8)
Laterality of the study eye, *n* (%)	Right Left	36 (56.3) 28 (43.8)	28 (59.6) 19 (40.4)

Abbreviation: SD, standard deviation; IOP, intraocular pressure; IQR, interquartile range; CDR, cup-to-disc ratio; logMAR, Logarithm of the Minimum Angle of Resolution.

**Table 2:** Test–retest reliability and absolute repeatability of MRF global visual field indices at baseline and 4 weeks (*n* = 47).

MRF Parameter	Baseline, Mean ± SD	Week 4, Mean ± SD	Mean Difference *	95% LoA ^†^	CoR ^‡^	ICC (95% CI)
MD (dB)	−6.31 ± 5.74	−6.45 ± 5.45	−0.14	−2.00 to 1.72	1.86	0.993 (0.987–0.996)
PD (dB)	7.58 ± 3.34	7.78 ± 3.29	0.20	−0.89 to 1.29	1.09	0.992 (0.985–0.996)
VC (%)	81.92 ± 17.44	81.93 ± 16.44	0.01	−6.06 to 6.08	6.07	0.992 (0.985–0.995)

Notes: * Mean difference calculated as the week 4 measurement minus the baseline measurement. ^†^ 95% limits of agreement (LoA) calculated as mean difference ± 1.96 × SD of the paired differences. ^‡^ Coefficient of repeatability (CoR) calculated as 1.96 × SD of the paired differences. Abbreviation: MRF, Melbourne Rapid Fields; SD, standard deviation; LoA, limits of agreement; CoR, coefficient of repeatability; ICC, intraclass correlation coefficient; CI, confidence interval; MD, mean deviation; PD, pattern deviation; VC, visual capacity.

**Table 3:** Comparison and agreement of visual field indices between MRF and HFA.

Parameter	MRF, Mean ± SD	HFA, Mean ± SD	Mean Difference (HFA-MRF)	ICC (95% CI)	^a^ *p*-Value
MD, dB	−6.27 ± 5.55	−8.32 ± 5.47	−2.05	0.949 (0.916–0.969)	**<0.001**
PSD/PD, dB	7.48 ± 3.26	6.33 ± 4.11	−1.15	0.880 (0.802–0.927)	**<0.001**
VFI/VC, %	82.19 ± 17.19	82.45 ± 16.36	0.26	0.930 (0.884–0.957)	0.808

Notes: ^a^ paired-samples *t*-test. Significance is marked in bold (*p* < 0.05). Abbreviations: MRF, Melbourne Rapid Fields; HFA, Humphrey Field Analyzer; SD, standard deviation; ICC, intraclass correlation coefficients; CI, confidence interval; MD, mean deviation; dB, decibels; PSD, Pattern Standard Deviation; PD, Pattern Deviation; VFI, Visual Field Index; VC, visual capacity.

**Table 4:** Agreement between MRF and HFA visual-field indices according to glaucoma severity.

Parameter	Severity	*n*	Mean Difference, HFA − MRF	95% LoA	^a^ Proportional Bias, *p*
MD (dB)	Mild	25	−1.22	−4.08 to 1.64	0.415
	Moderate	24	−2.53	−5.15 to 0.09	**<0.001**
	Severe	15	−2.69	−8.65 to 3.27	**0.001**
PSD/PD (dB)	Mild	25	−2.19	−6.03 to 1.65	0.055
	Moderate	24	−0.93	−6.36 to 4.50	0.171
	Severe	15	0.25	−3.24 to 3.74	0.912
VFI/VC (%)	Mild	25	0.73	−7.03 to 8.49	0.587
	Moderate	24	0.84	−18.86 to 20.54	**0.019**
	Severe	15	−1.45	−24.28 to 21.38	0.064

Notes: ^a^ Proportional bias was obtained using linear regression within the Bland–Altman analysis. Significance is marked in bold (*p* < 0.05). Abbreviations: HFA, Humphrey Field Analyzer; MRF, Melbourne Rapid Fields; LoA, limits of agreement; MD, mean deviation; dB, decibels; PSD, Pattern Standard Deviation; PD, Pattern Deviation; VFI, Visual Field Index; VC, Visual Capacity.

**Table 5:** Patients’ acceptability on MRF test.

Questions	Answers	Frequency (%)
Easy to do	No Yes	0 (0.0) 64 (100.0)
Comfortable to do MRF test	No Yes	0 (0.0) 64 (100.0)
Stress to do MRF test	No Yes	64 (100.0) 0 (0.0)
Willing to undergo MRF testing again	No Yes	1 (1.6) 63 (98.4)
Has iPad at home	No Yes	63 (98.4) 1 (1.6)
Feels the same with HFA test or not	Feels both HFA and MRF test was equally similar	1 (1.6)
	Preferred MRF over HFA	63 (98.4)

Abbreviations: MRF, Melbourne Rapid Fields; HFA, Humphrey Field Analyzer.

## Data Availability

The data presented in this study are available on request from the corresponding author due to privacy and ethical restrictions.
